# Lead Fractures in Deep Brain Stimulation during Long-Term Follow-Up

**DOI:** 10.4061/2010/409356

**Published:** 2009-12-13

**Authors:** Fernando Seijo Fernández, Marco Antonio Alvarez Vega, Aida Antuña Ramos, Fernando Fernández González, Beatriz Lozano Aragoneses

**Affiliations:** ^1^Department of Surgical Neurology, Functional Neurosurgery Unit, University Central Hospital of Asturias, 33006 Oviedo, Spain; ^2^Department of Clinical Neurophysiology, University Central Hospital of Asturias, 33006 Oviedo, Spain

## Abstract

The purpose was to determine the incidence of lead fracture in patients with DBS over a long period of time. We present a retrospective study of 208 patients who received 387 DBS electrodes. Fourteen patients had sixteen lead fractures (4% of the implanted leads) and two patients suffered from 2 lead fractures. Of all lead fractures, five patients had the connection between the leads and the extension cables located in mastoids region, ten in cervical area and one in thoracic region. The mean distance from the connection between the electrode and the extension cable and the lead fracture was 10.7 mm. The lead fracture is a common, although long-term complication in DBS surgery. In our experience, the most common site of electrode cable breakage is approximately between 9 and 13 mm from the junction between the lead and the extension cable. The most important cause of lead fracture is the rotational movement of the lead-extension cable system. If we suspect lead fracture, we must check the impedance of the electrode and to evaluate the side effects of voltage. Finally, we must conduct a radiological screening.

## 1. Introduction

Deep brain stimulation (DBS) utilisedsuccessfully in movement disorders and pain is not exempt from complications. Thus, the incidence of hardware-relatedcomplications affects 13.9% of patients, who underwent surgery [1] and 4.3%–8.4% ofimplanted electrodes per year [2, 3]. The most common complications and percentages are infection in 6.1% of patients, migration or misplacement of the leads in 5.1% of patients, skin erosion in 1.3% of patients and lead fractures in 1%–15% of patients [4–8]. In this article, we will review the long-term incidence of lead fractures in DBS surgery on a total of 387 DBS electrodes implanted from 1996–2007.

## 2. Objective

Our objective is to determine the long-term incidence of lead fractures in patients who underwent DBS surgery.

## 3. Methods

This article is a retrospective study, 1996–2007, on a consecutive series of patients who underwent DBS surgery. All patients were operated on by the same surgeon (F.S.) at the same hospital and with the same surgical methodology [9].

Out of a total of 208 patients, 387 DBS electrodes (3387/3389 Medtronic Inc, Minneapolis, MN, USA) were implanted, with 179 patients who received a bilateral implant and 29 who received a unilateral implant. The patients' diagnoses were Parkinson's disease, tremors, dystonias, and cluster headache. The DBS electrodes were implanted in the thalamus, internal pallidus, subthalamic nucleus, and hypothalamus.

The connection site between the DBS electrode and the extension cable was the cervical region in 264 connections, the mastoid region in 75, and the thorax in 34. The mean followup period of all cases was 51 months (Range: 6–125). The length of the DBS electrodes depended upon the type of internal pulse generator (IPG) (Soletra or Kinetra, Medtronic Inc, Minneapolis, MN, USA). The majority of connections that were performed in the thorax pertained to the Kinetra IPG.

Three patients died during this period due to causes unrelated to the surgery and four patients did not keep in contact with us. All these patients had received the DBS electrode implanted bilaterally and were not included in this article. 

## 4. Results

**Figure 1:**
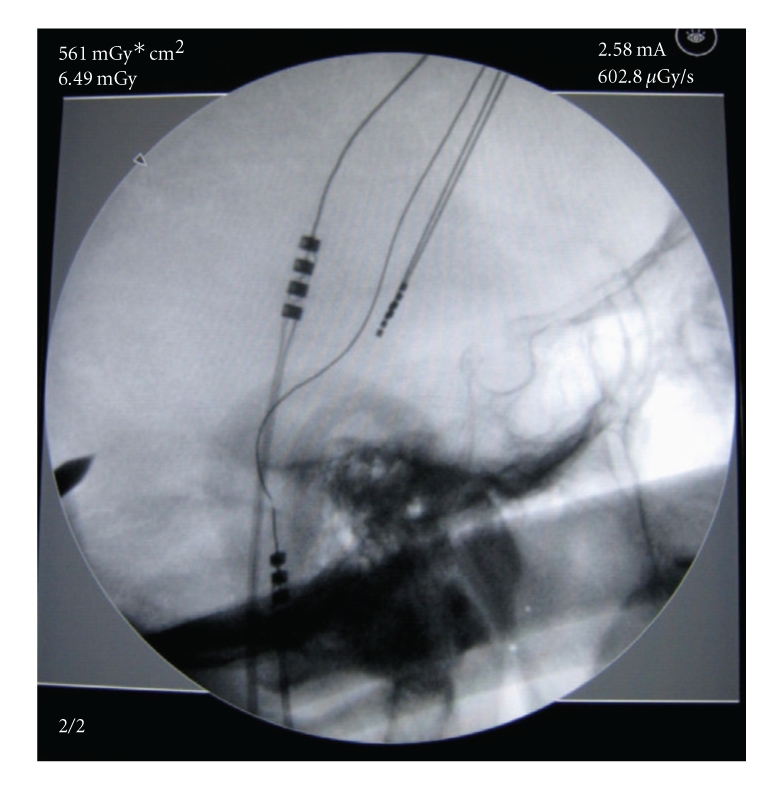
Lead Fracture with connection located in mastoid region.

**Figure 2:**
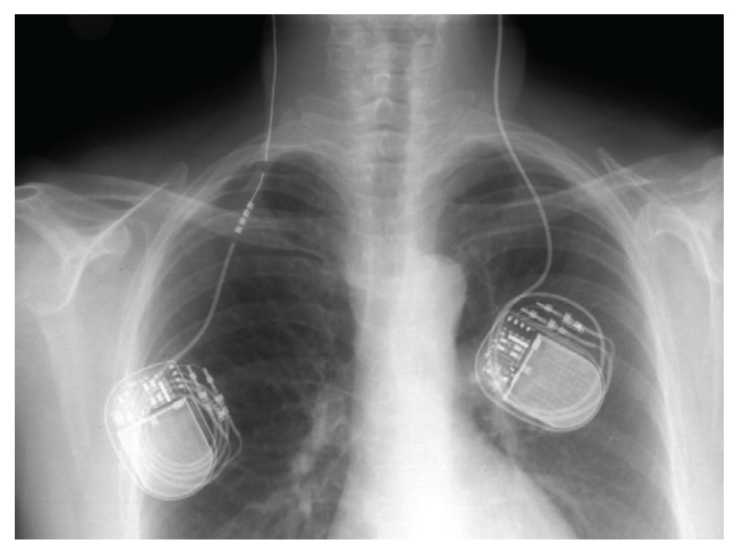
Lead fracture with connection located in thoracic region.

**Figure 3:**
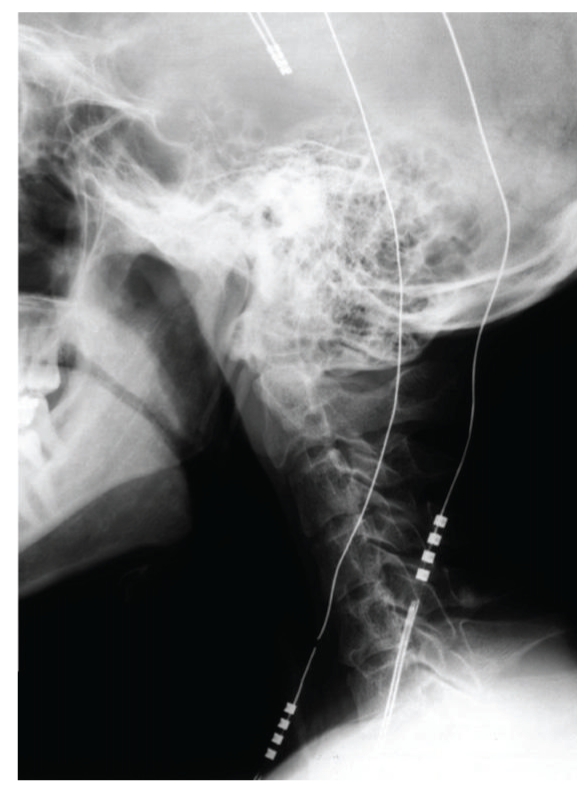
Lead fracture with connection located in cervical region.

Between June of 1996 and December of 2007, 22 patients (10.5% of all patients) had complications related to the hardware. These complications were 16 lead fractures, 4 erosions, and 2 infections in the IPG. Out of 22 patients who had complications related to the hardware, fourteen patients, 9 men and 5 women, had 16 lead fractures (4% of the implanted leads) and two patients suffered from 2 lead fractures. The mean age of these patients was 57 years (Range: 28–75 years). The lead fractures were localised with 10 in the cervical region, 5 in the mastoid region, and 1 in the thorax (Figures 1, 2, and 3). The first diagnosis of lead fracture was in October of 2001 and the last in November of 2008. The mean time between the DBS surgery and the diagnosis of lead fracture was 36 months (Range: 7–84 months) and the mean distance from the connection between the electrode and the extension cable and the lead fracture was 10.7 mm (range: 9–13.2 mm); see Table 1.

## 5. Discussion

According to the data obtained in literature, the breakage of leads and wires is present in 5% of patients who underwent DBS surgery and in 1.8% of implanted electrodes [4]. We present sixteen lead fractures (4% of the lead implants) in fourteen patients (7% of all cases). In other words, in our experience with 387 implanted electrodes, the most common hardware-related complication was lead fracture. 

**Figure 4:**
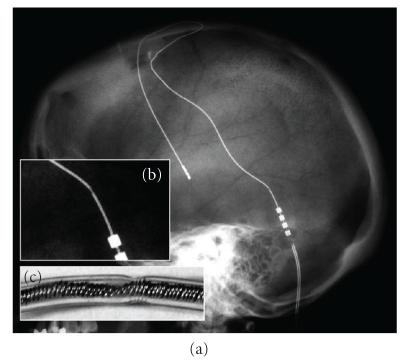
Incomplete lead fracture. (a) Rx of skull. (b) Detail of incomplete lead fracture. (c) A distortion of the explanted lead of (a) is observed.

**Figure 5:**
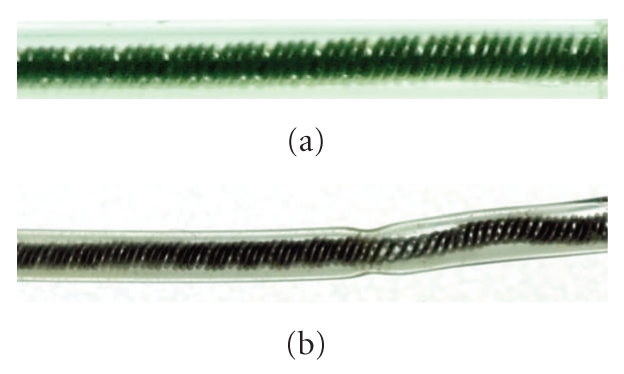
(a) Normal Lead. (b) We observed an explanted lead different from Figure 4.

In the case of 12 patients with Parkinson's disease, these patients suffered from an abrupt worsening of their disease: in the case of the patient with cluster headache, the pain returned, and in the case of essential tremor, it reoccurred. In the followup examination, impedance as well as current was measured. In 14 electrodes, impedance exceeded 2000 ohms and two were normal, and in the 16 electrodes checked, no side effects occurred with voltages of 10.5 V. For the purpose of confirming electrode breakage, all patients underwent X-rays and in all cases lead fracture was observed (Figures 1, 2, and 3). In the two cases where impedance was normal on the X-ray of the lead, a change in its structure was observed but without attaining a complete fracture (Figures 4 and 5). Whereas the majority of the authors [2, 6, 10] describe this finding between 6 and 24 months after implanting the electrode, in our case, it was detected at about 36 months (Range: 7–84 months). 

It is surprising to see that none of the 4 patients with dystonia (out of a total of 208 patients) who underwent DBS surgery experienced electrode breakage [11]. We think that this event is due to the fact that in two cases the result was spectacular and the patients no longer experienced the stereotyped movements typical of dystonia, and in another case, because the dystonia was focal and only affected the upper right extremity, its movements did not have an influence on the trajectory of the lead, and in the fourth case, because even though the movements due to dystonia did not disappear, they did decrease and this put the patient in the same range of electrode breakage as those patients who suffered from Parkinson's disease and who undergo DBS surgery. 

 Why did the breakage fracture always occur at 10.7 mm (range 9–13.2) from the connection between the lead and the extension cable? In the study that we conducted, we observed the following events out of all our cases: (i) All patients who underwent surgery showed a wide loop in the lead at the cranial level. This was done to avoid displacement of the electrode at a cerebral level. (ii) Lead fractures are not related to the type of IPG (Soletra or Kinetra, Medtronic Inc, Minneapolis, MN, USA) or to the type of lead (3387/3389, Medtronic Inc, Minneapolis, MN, USA) or to the length of the extension cable. (iii) The mean time of breakage was long, 36 months. We think that cervical movements transmit flexion-extension movements, but above all, rotation to the extension cable and this transmits those movements to the lead, with the point of maximum force being between 9 and 13 mm from the connection between the extension cable and the lead (Figures 4 and 5). The extension cable could become twisted with movements without breaking because of its greater resistance whereas the lead, being more fragile and anchored to the cranium, would be more affected by the force of these movements. Normal material fatigue with the passage of time must also be kept in mind [12].

The connection between the lead and the extension cable at a cervical level has been associated with a greater incidence of wire fractures [2]. However, in our experience, the percentage rate for breakage fractures in regards to the connection site was at 6.5% (5 cases of 75) in the mastoid region, 3.8% (10 cases of 264) in the cervical region, and 3% (1 case of 34) at the thoracic level. The majority of our connections between the lead and the extension cable were carried out in the cervical region to avoid erosions since they occur more frequently if this procedure is carried out on a hard surface such as the cranium (4). None of our patients in whom the connection was carried out between the lead and the extension cable in the cervical region presented skin erosions.

## 6. Conclusion

Lead fracture is a common, although long-term complication in DBS surgery. In our experience, the most common site of electrode cable breakage is approximately between 9 and 13 mm from the junction between the lead and the extension cable. We believe that the most important cause of lead fracture is the rotational movement of the lead-extension cable system. If we suspect lead fracture, we must check the impedance of the electrode and to evaluate the side effects of voltage. Finally, we must conduct a radiological screening. 

## Figures and Tables

**Table 1:** C1, C2, C3, C5, C6: cervical vertebral body. CL: cluster headache. DBS: deep brain stimulation. H: hypothalamus. M: male. F: female. PD: Parkinson's disease. STN: subthalamic nucleus. T1: thoracic vertebral body. VIM: ventralis intermediate nucleus.

Patient/Sex	Diagnostic/ Place of implant DBS	Age	Date of implant	Date of break	Place of break	Distance of break (mm)	Conexion model
1M	Tremors/VIM	28	01/20/1998	10/03/2001	C5	12.1	7495
2M	E.P/ NST	63	05/26/2001	04/22/2008	C3	9.2	7495
3F	E.P/NST	54	05/22/2003	02/01/2006	T1,	13.22	7489
			05/25/2006	01/10/2007	mastoid	9.9	7489
4F	E.P/NST	56	06/24/2004	06/01/2006	C5	9.7	7489
5F	E.P/NST	69	04/14/2005	01/03/2007	C1,	11.30	7489
			04/17/2007	02/21/2008	mastoid	9.83	7489
6F	CL/H	47	02/19/2006	09/04/2007	C6	13.3	7489
7M	E.P/NST	40	07/05/2006	01/16/2008	C5	12.39	7489
8M	E.P/NST	75	03/23/2003	04/11/2007	C2	9.44	7489
9M	E.P/NST	59	02/05/2004	09/10/2008	mastoid	11.68	7489
10M	E.P/NST	59	06/14/2007	09/22//2008	mastoid	9.52	7489
11F	E.P/NST	72	06/24/2003	10/15/2007	C5	9.61	7489
12M	E.P/NST	60	04/11/2002	09/10/2007	C2	9	7489
13M	E.P/NST	61	05/19/2005	11/19/2008	C2	8.2	7489
14M	E.P/NST	58	11/03/2005	06/24/2008	mastoid	8.8	7489
